# Agricultural Eco-Efficiency Response and Its Influencing Factors from the Perspective of Rural Population Outflowing: A Case Study in Qinan County, China

**DOI:** 10.3390/ijerph20021016

**Published:** 2023-01-05

**Authors:** Yanling Zong, Libang Ma, Zhihao Shi, Min Gong

**Affiliations:** 1 College of Geography and Environmental Science, Northwest Normal University, Lanzhou 730070, China; 2 Key Laboratory of Resource Environment and Sustainable Development of Oasis, Lanzhou 730070, China; 3 Institute of Urban and Rural Development and Collaborative Governance of Northwest, Lanzhou 730070, China

**Keywords:** population outflow, non-agricultural level of labor force, aging, income, agricultural eco-efficiency, spatial spillover

## Abstract

Agriculture is the source of human clothing and food, but it also brings negative externalities to the environment. The outflow of the rural population is one of the factors for changes in the characteristics of the rural population. Farmers’ decisions on agricultural production can affect agricultural ecological efficiency. Therefore, it is necessary to study the relationship between the two in rural development. Taking Qin’an County in the Loess Hilly Region of central Gansu, China, as an example, this paper analyzed the demographic characteristics and the evolution characteristics of agricultural eco-efficiency under the background of rural population outflowing, and the impact of the former on the latter, based on the panel statistical data of 17 villages and towns from 2001 to 2020. The results show that (1) From 2001 to 2020, the non-agricultural level of Qin’an County’s labor force showed an upward fluctuation trend. The level of aging was relatively stable, and the per capita disposable income was significantly increased. (2) From 2001 to 2020, the agricultural eco-efficiency of Qin’an County showed a wavy change, but there were some towns and villages that have not been effectively developed. The regional differences are significantly different. (3) The non-agriculturalization level of the labor force promotes agricultural eco-efficiency through the direct effect rather than the space spillover effect. The positive effect of aging on agricultural eco-efficiency was mainly reflected through direct effect rather than spatial spillover effect. Per capita, disposable income has a significant positive spatial spillover effect on agricultural eco-efficiency. Finally, this paper provides a scientific reference for promoting the improvement of agricultural eco-efficiency and sustainable development. This is of great theoretical and practical significance for the realization of rural revitalization.

## 1. Introduction

The rapid development of economic globalization and international trade has brought serious pollution problems to the global environment. The eco-efficiency has attracted great attention from governments and scholars all over the world. As one of the sources of carbon emissions, carbon emissions from agriculture accounts for 11% of the global emissions, and only 7% in the United States, but up to 17% in China [1], that’s more than double the global rate and nearly three times the U.S. rate, this shows that the level of carbon emissions from agriculture is not optimistic. Therefore, it is important to improve the mode of agricultural development for environmental protection. The green development goal of China’s agricultural sector has a long way to go. As a national basic industry, agriculture plays a vital role in national stability and food security.

Over the past 40 years of reform and opening up in China, comprehensive agricultural production capacity and farmers’ income have continued to increase. The growth rate of farmers’ income has been greater than that of urban residents’ income for five consecutive years and has achieved great achievements [2]. However, according to the estimate of the Asian Development Bank, the direct economic loss caused by the destruction of China’s agricultural resources and the environment accounts for about 1% of the national GDP [3]. In addition, due to the excessive application of chemical fertilizers and pesticides, a large number of agricultural exports suffer from green trade barriers every year. It is difficult to estimate the indirect loss [4], and a series of problems arose. With the intensification of negative effects and the growing concern for global sustainable development, the promotion of agricultural ecological development has attracted more attention. China’s agriculture has gradually ushered in a critical moment for the return of essential functions and transformation and upgrading.

With the continuous development of industrialization and urbanization, the rural population has continued to outflow, and a large number of rural labor forces have been separated from agriculture. According to the 2020 Monitoring Survey report on Migrant Workers released by China’s National Bureau of Statistics, by the end of 2020, the total number of migrant workers in China had reached 285 million. The outflow of the rural population, especially the outflow of young people, has led to serious rural diseases of rural areas characterized by the hollowing out of the countryside and the aging and weakening of social subjects. This has brought great challenges to rural society and the economy [5]. Population plays an important role in rural development. The dynamic behavior of the population in space not only directly affects the change of rural population characteristics but also has a radiation effect on the surrounding areas through capital flow, logistics, information flow, etc. This leads to spatial spillovers in all aspects of the countryside, and agricultural production is one of them. Agricultural ecologicalization belongs to the category of industrial ecologicalization, which is a good method of development. Its goal is to promote sustainable agricultural development. The focus is to comprehensively balance the relationship between agricultural input, agricultural output, and ecological impact. The basic index to measure the development level of agricultural ecologicalization is agricultural eco-efficiency, and eco-efficiency is now widely used in the agricultural sector [6].

Agricultural ecological efficiency refers to obtaining as much agricultural output as possible with as little resource consumption and environmental pollution as possible under a certain combination of agricultural input elements [7]. Researchers have carried out many empirical studies on agricultural eco-efficiency with in-depth discussions from macro, meso, and micro levels [7,8,9,10]. Their measurement methods mainly include stochastic frontier analysis and data envelopment analysis methods, super-efficiency DEA, three-stage DEA, and undesirable SBM models [7,11,12]. Scholars have studied the spatiotemporal characteristics and driving factors of agricultural eco-efficiency in different regions. Foreign scholars mainly measure the eco-efficiency and influencing factors of the agricultural system by constructing a comprehensive indicator system. They found that the use of chemical fertilizers and pesticides can reduce agricultural eco-efficiency. The degree of environmental protection of the farm is positively correlated with agricultural production efficiency [10,13,14]. Most scholars in China use data envelopment analysis, stochastic frontier analysis methods, and undesirable SBM models to analyze the agricultural eco-efficiency from the macro, meso, and micro scales, the spatial-temporal characteristics of agricultural eco-efficiency and its influencing factors [15] as well as improving paths [16], low-carbon development [17,18] and the mechanism [8]. Some scholars have studied the interaction between rural labor and non-agricultural level, the intensity of fertilizer application, and agricultural eco-efficiency [19]. In terms of unexpected agricultural output, existing studies usually input agricultural surface source pollution [20] or agricultural carbon emissions [16] or these two unexpected outputs [8] into the model. Based on the availability of data, this paper takes agricultural carbon emissions into account.

In sum, there are many studies on agricultural eco-efficiency, but there are still some limitations. First, scholars mainly focus on the research of agricultural eco-efficiency on the macro-scale [19] and meso-scale [17]. There are few studies at the micro-scale, especially at the township scale. There are differences in the agricultural economic level, factor input, resource endowment, and location conditions in different regions; large-scale studies do not reflect the reality in small areas. Therefore, it is necessary to conduct more research on the micro-scale. Second, most of the existing studies mainly focused on the characteristics of the agricultural eco-efficiency in a certain area, and there are few studies from the perspective of population outflowing. Taking agriculture in a narrow sense as the research object, this paper put forward the assumptions of the relationship between population and agricultural eco-efficiency from the theory of farmers’ behavior. In addition, this paper studied the relationship between the rural population and the agricultural eco-efficiency with the SDM based on the micro statistics from towns and towns, and the following conclusions were drawn: The promotion effect of non-agricultural labor level on agricultural eco-efficiency was mainly reflected through direct effect rather than spatial spillover effect; The promoting effect of aging on agricultural eco-efficiency was mainly reflected through direct effect rather than spatial spillover effect. Per capita disposable income has a significant positive spatial spillover to agricultural eco-efficiency. The rural population is the main body of agricultural production and direct user of cultivated land; the decision-making behavior of ecological farming directly influences the quality of cultivated land and the development of rural agriculture, and with the development of urbanization, the population outflow, agricultural development is the greater challenge; in this case, more needs to be based on the research of population outflow to provide a related reference for policymakers and, in turn, make better policy to improve the agricultural environment, promote rural development. Therefore, the study not only can enrich the research content on farmers’ decision-making behavior but can also expand the decision-making behavior of peasant household ecological farming research depth.

## 2. Literature Review

Agriculture is the basic industry of the Chinese national economy. With the advancement of agricultural technology in China, the level of agricultural production has been significantly improved. In addition, agricultural pollution from agricultural production is increasing in China. Therefore, it is urgent to improve the environment for agricultural development. Promoting green agricultural development is the only way to transform agricultural development and promote the transformation and upgrading of the agricultural industry. This requires agricultural production to minimize the negative externalities of the environment [21]. Therefore, improving agricultural eco-efficiency is the key to achieving sustainable development and a win-win for the economy and environment in China [22]. The problems of rural population outflow, the lack of social subjects, aging, and the deterioration of grass-roots organizations are more serious [5]. In addition, it has promoted the rapid growth of the rural economy, enriching the income source of farmers [7]. The characteristics of the rural population have changed with the population outflow. The labor force has shifted from the agricultural sector to the non-agricultural sector, the aging has increased, and the per capita disposable income has fluctuated. Those are reflected in the reduction of the total labor force, the decline of physical fitness, the decline of labor capacity, the diversity of agricultural allocation, and the rich source of farmers’ income. Agricultural production is one of the behaviors of farmers. There is a one-to-one relationship between farmers and rational persons in economics, and farmers are typical risk avoiders. They often make decisions based on the maximization of family benefits, and their production purpose is to meet the needs of survival and living. Therefore, they will weigh them according to their actual needs and make more reasonable choices. From the perspective of China’s rural population, farmers decide to conduct agricultural production based on the benefits and the impact on their family’s production and life. This can change their input-output level in agricultural production. Therefore, this paper analyzed the relationship between them from the perspective of rural population outflow.

The rural population continues to move to the city under urbanization. There are three mechanisms for the rural population in the development of agriculture. First, the reduction of the total labor force caused by population outflow directly affects the input of the labor force in agricultural production, resulting in changes in agricultural industries [16]. Second, population outflow leads to the improvement of agricultural development conditions such as agricultural Technology. This affects the input and output of agricultural production, thereby indirectly affecting agricultural industry [16]. The third is that the rural population not only directly affects output as an input factor of agricultural production but also indirectly affects agricultural production through other aspects such as mechanization and chemical substances. The three mechanisms have different paths, and the latter two reflect the spillover effect of population characteristics. First, the transfer of non-agricultural populations in the rural population releases the rural surplus labor force [7], resulting in the reallocation of family labor. This affects the labor force input in agricultural production and directly affects the agricultural eco-efficiency. Second, the mechanism of labor non-agricultural transfer and aging is the reduction of the total labor supply [16], thereby reducing the pressure of the population on the agricultural ecological environment and strengthening the self-regulation function of the ecosystem. With the development of agricultural modernization, the input of the agricultural labor force has decreased, leading to the reform and innovation of agricultural production technology and the surrounding imitation effect to a certain extent. Farmers can replace the labor force through agricultural mechanization. The increase in per capita disposable income can lead to an increase in agricultural capital investment, for example, by increasing the input of chemical fertilizers and pesticides to avoid the loss caused by disasters. However, it can increase agricultural carbon emissions, thereby indirectly affecting agricultural production efficiency. Third, the rural population is the labor force engaged in agricultural production. The dynamic behavior of the rural population directly affects the input of the agricultural production labor force with a series of spillover effects, such as the fluctuation of production technologies such as mechanization and agricultural funds. This leads to a change in agricultural input and output and then affects the agricultural eco-efficiency. Therefore, changes in the characteristics of the rural population are closely related to agricultural eco-efficiency. The former is the direct influence factor with spillover effects. With the non-agricultural transfer, aging, and the increase in income, farmers will give up agricultural production or change their planting structure after weighing human capital. They are more inclined to plant crop that is easy to cultivate, with high yield and strong adaptability to mechanization [23]. These are a series of decisions that lead to changes in agricultural production, thereby changing agricultural eco-efficiency.

**Figure 1:**
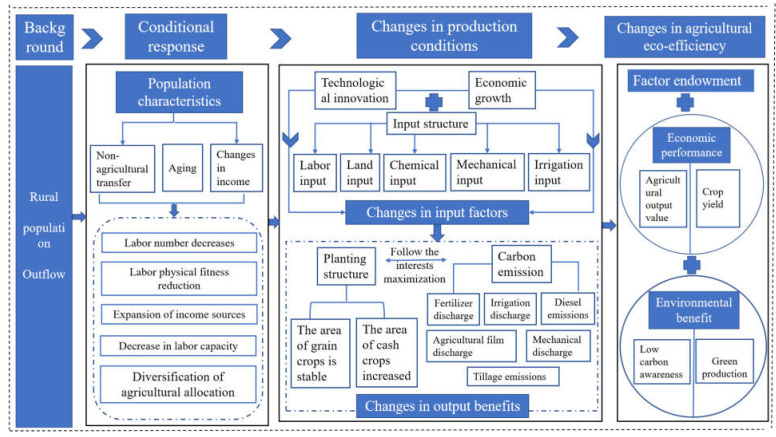
The analytical framework of agricultural eco-efficiency under the change of rural population.

In sum, farmers can make a rational decision based on the conditions of production factors such as family labor, capital, land, and market changes. Population outflow caused a series of changes in demographic characteristics resulting in changes in the endowment of agricultural production factors, agricultural output and output value, and agricultural carbon emissions. The continuous fluctuations of factor endowment, economic benefits, and environmental benefits lead to changes in agricultural eco-efficiency. Under the demand of changes in factor endowment, economic benefits, and environmental benefits, the rural population comprehensively measures the input-output elements and makes the most favorable decisions, finally responding to agricultural eco-efficiency (Figure 1).

## 3. Overview of the Study Area and Data Sources

### 3.1. Overview of the Study Area

#### 3.1.1. Natural Geographical Overview

**Figure 2:**
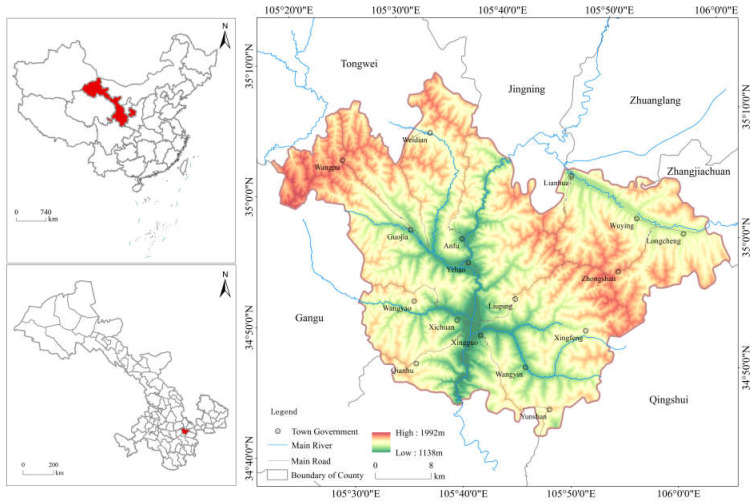
Location of Qinan County.

Qin’an County is located in the southeast of Gansu Province, China, in the Loess Plateau, north of Tianshui City, and the lower reaches of the Hulu River in the Weihe River. It is located between 105°21′~106°02′ E and 34°44′~35°11′ N, with a total area of 1604.01 km^2^, as shown in Figure 2. There are many mountains in the county, undulating beams, and ravines. It is a typical loess hilly and gully area. The climate is mild, the sunshine is sufficient, the rainfall is less, and the drought is frequent. It belongs to the semi-humid monsoon climate region. The complex terrain conditions and climate lead to a difference in the input of agricultural factors in Qin’an County, thereby forming the north-south difference in agricultural eco-efficiency.

#### 3.1.2. Overview of Socio-Economic

Qin’an County is a node city of the national “Belt and Road” initiative and the planning of the Guanzhong Plain urban agglomeration. There are 17 townships, 428 administrative villages, and eight communities. In 2020, the total number of rural labor resources in the county was 365,344, of which 199,642 were non-agricultural labor, accounting for 54.64% of the total rural labor. The GDP was 7.938 billion yuan, of which the added value of the first production was 2.084 billion yuan, a year-on-year increase of 5.7%. Agriculture in Qin’an County is dominated by food crops and cash crops. Due to the high benefits of cash crops, the area of cash crops has increased year by year. In 2020, the area of cash crops in the county reached 9364.91 acres. In 2020, the county’s agricultural fertilizer was 59,402 tons, pesticide usage was 2796 tons, and the amount of plastic film was 916.07 tons. In 2020, the number of the non-agricultural labor force in Qin’an County reached 199,462, accounting for 34.28% of the total population of the county. The aging rate reached 14.19%. The rural per capita disposable income was 9086 yuan. With the process of urbanization, the conditions and sources of income for labor forces to work have been enriched, which has led to the diversification of their decisions. This has caused agricultural eco-efficiency to a certain extent.

### 3.2. Data Sources

This study took 17 villages and towns in Qin’an County in the Loess Holo Area as the evaluation unit and 2001–2020 as the research period. The data are mainly from 2 sources: (1) Basic diagram: Qin’an County vector administrative boundary (1: 250,000) was from the Tianshui Natural Resources Bureau; DEM grid data in Qin’an County were from geographical space data cloud; (2) Township statistics: From 2001 to 2020, the population data of towns and towns in Qin’an County (non-agricultural employment, 60-year-old and above population data, per capita disposable income), agricultural input factor (total crop seeding area, agricultural practitioners, agricultural total machinery power, fertilizer application amount, agricultural film application amount, pesticide use, effective irrigation area), output element (grain crop yield, cash crop yield, total agricultural output value, fertilizer, pesticide, agricultural film and diesel use) and influencing factors (the area of cultivated land, the area of cash crops, the affected area, and the agricultural output value and the total agricultural output value) were from the *Qin’an County Statistical Yearbook (2001–2021)*. The missing data were obtained by interpolation. The relevant data on the general situation of the study area are all from the 2021 *Qinan County Statistical Yearbook*.

## 4. Method

### 4.1. The Super-SBM Model

Eco-efficiency is a coordinated relationship between resource and environmental input and human activity production, that is, a balance between economic and environmental. Tone proposed a super-efficiency SBM model (super slack-based measure) to avoid problems such as the deviations and effects caused by different dimensions and radial and angle selection differences [24]. It is widely used in the evaluation of eco-efficiency. It can not only distinguish expected and unexpected outputs but also facilitates the optimization of eco-efficiency. Therefore, this paper took agricultural carbon emissions as an unexpected output and used the Super-SBM model to calculate the eco-efficiency of the agriculture. The formulas are

(1) $$min\delta =\frac{1+\frac{1}{m}\sum_{i=1}^{m}\frac{s_{i}^{-}}{x_{ik}}}{1-\frac{1}{q_{1}+q_{2}}(\sum_{r=1}^{q_{1}}\frac{s_{r}^{+}}{y_{rk}}+\sum_{t=1}^{q_{2}}\frac{s_{t}^{b-}}{b_{tk}})}$$

(2) $$\overset{n}{\underset{j=1,j\neq k}{\sum }}x_{j}\lambda_{j}-s_{i}^{-}\leq x_{ik}$$

(3) $$\overset{n}{\underset{j=1,j\neq k}{\sum }}y_{j}\lambda_{j}+s_{r}^{+}\geq y_{rk}$$

(4) $$\overset{n}{\underset{j=1,j\neq k}{\sum }}b_{j}\lambda_{j}-s_{t}^{b-}\leq b_{tk}$$

(5) $$1-\frac{1}{q_{1}+q_{2}}(\overset{q_{1}}{\underset{r=1}{\sum }}\frac{s_{r}^{+}}{y_{rk}}+\sum_{t=1}^{q_{2}}\frac{s_{t}^{b-}}{b_{tk}})>0$$

(6) $$\lambda_{j},s_{i}^{-},s_{r}^{+},s_{t}^{b-}\geq 0$$

(7) $$i=1,2,\ldots ,m;r=1,2,\ldots ,q_{1};t=1,2,\ldots ,q_{2};$$

(8) $$j=1,2,\ldots ,n\left(j\neq k\right)$$

where *δ* is eco-efficiency, *j* is each DMU, $\lambda_{j}$ is a strength variable. $s_{i}^{-},\;s_{r}^{+},\;s_{t}^{b-}\;$ represent the relaxation variables of input, expected outputs and unexpected outputs, respectively.$\;x_{j},\;y_{j},\;b_{j}$ are the input-output variables of the *j*th decision-making unit. $x_{ik},\;y_{rk}$ and $b_{tk}\;$ represent the evaluation of the input, expected output, and unexpected output variables of $DMU_{k}$, respectively.

### 4.2. Spatial Econometric Models

Anselin [25] found that the economic phenomenon of any region does not exist in isolation, and there is a certain connection with its surrounding areas. The closer the geographical distance, the stronger the connection between regions. Two spatial autocorrelation models, the space lag model, and the spatial error model, have been established according to the idea. Lesage et al. [26] further constructed the spatial Durbin model. Based on the test and estimation of the spatial model, we used the spatial Durbin model. The expression of the model is

(9) $$\begin{array}{ll} Y=\alpha +\rho WY+ & \beta_{1}Rlne+\beta_{2}Income+\beta_{3}Age+\beta_{4}Land+\beta_{5}Ir+\beta_{6}Mci\\ & +\beta_{7}Cps+\beta_{8}Adr+\beta_{9}As+\theta_{1}WRlne+\theta_{2}Income+\theta_{3}Age\\ & +\theta_{4}Land+\theta_{5}Ir+\theta_{6}Mci+\theta_{7}Cps+\theta_{8}Adr+\theta_{9}As+\varepsilon \end{array}$$

where *Y* is explained variables. α is the constant item. ρ is the spatial lag regression coefficient. *W* represents the space distance weight of the area. β is the regression coefficient of explanatory variables. θ represents the space lags of the variables. ε is a random disturbance item that is independent and identically distributed.

### 4.3. Variable Selection

#### 4.3.1. Input-Output Indicator

According to agricultural production practice and previous research [8], this paper selected 7 input indicators, as shown in Table 1. Land input: the total planting area of crops; Labor input: agricultural practitioners; Fertilizer input: the amount of rural fertilizer application; Pesticide and agricultural film input: there are many types of pesticides and a wide range of applications. It is difficult to refine it in macro evaluation. According to existing research, pesticide input is characterized by the amount of pesticide used. Agricultural file input is characterized by the amount of agricultural film used; Agricultural machinery power input: mechanization is an important feature of modern agriculture. The total power data of agricultural machinery are from statistical data. Irrigation input: the effective irrigation area.

According to previous studies, the expected agricultural output index is represented by total agricultural output value [16] and crop yield [8] (including cash crops and grain crops), as shown in Table 1. The source of agricultural carbon emissions mainly includes the use of agricultural chemicals, the consumption of fossil fuels by agricultural machinery, the indirect emissions caused by the consumption of electricity for agricultural irrigation, and the loss of organic carbon caused by agricultural farming. Therefore, this article used chemical fertilizer, pesticide, agricultural film, agricultural diesel, agricultural irrigation, and agricultural farming as indicators to estimate the amount of agricultural carbon emissions [8]. According to existing research, the emission coefficients of the above six types of carbon emissions are as follows: fertilizer 0.896 (kg/kg), pesticide 4.934 (kg/kg), agricultural film 5.180 (kg/kg), diesel fuel 0.593 (kg/kg), agricultural irrigation 20.476 (kg/hm^2^), agricultural farming 312.600 (kg/hm^2^).

#### 4.3.2. Influential Factor Indicators

Agricultural eco-efficiency is affected by population and income factors, and various factors such as resource endowment and natural conditions. This paper studied the spatial effect of population characteristics on agricultural eco-efficiency under the population outflow. In this paper, agricultural eco-efficiency is the dependent variable. The non-agricultural level of labor, aging, and per capita disposable income are the core independent variables. In addition, factors such as resources and environment that affect agricultural eco-efficiency are introduced into the model as control variables. The agricultural eco-efficiency is the explanatory variable, calculated through the super-efficiency SBM model. We used the level of non-agriculturalization, aging level, and per capita disposable income to characterize the population characteristics for core explanation variables. Because agricultural eco-efficiency is the result of many factors, this paper mainly used land scale, irrigation index, re-seeding index, planting structure, agricultural affected rate, and agricultural structure on the basis of population characteristics to characterize control variables, as shown in Table 2. The reason why these factors are considered is mainly determined on the basis of previous studies and theoretical demonstrations. Existing studies have shown that agricultural technology, public investment [27], and human investment [28] can all have a significant impact on agricultural production. In addition, agricultural planting structure [27] and agricultural disaster rate [16] will both affect the agricultural output value and the degree of factor input, so it has also been proved to have an impact on agricultural ecological efficiency. The research also showed that the higher the rate of land multiple cropping, the higher the utilization of agricultural land, and the more beneficial to the improvement of agricultural ecological efficiency [22].

## 5. Results and Discussion

### 5.1. The Changing of Rural Population with Population Outflow

**Figure 3:**
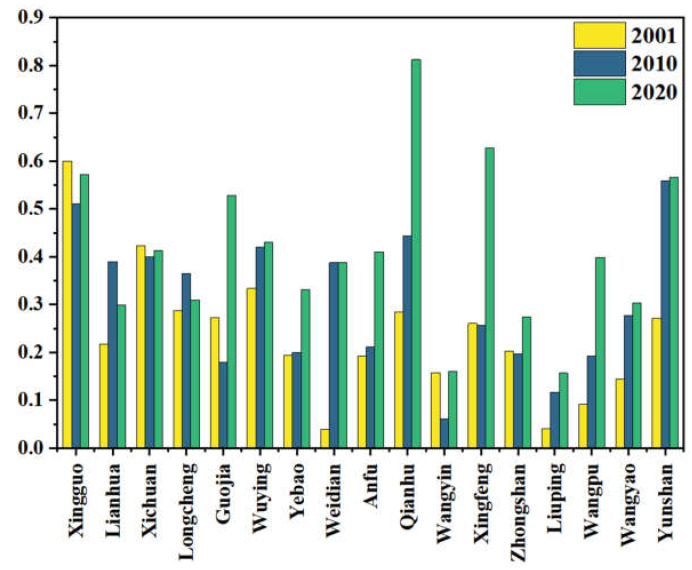
The level of non-agricultural labor force in Qin’an County.

Under the background of population outflowing, the rural population characteristics of Qin ’an County changed significantly. The non-agricultural level of labor force showed a fluctuating upward trend. There were 17 villages and towns with non-agricultural labor transfer. The average value was increased from 0.2362 in 2001 to 0.4107 in 2020. The overall change in the south was lower than that in the north, as shown in Figure 3.

**Figure 4:**
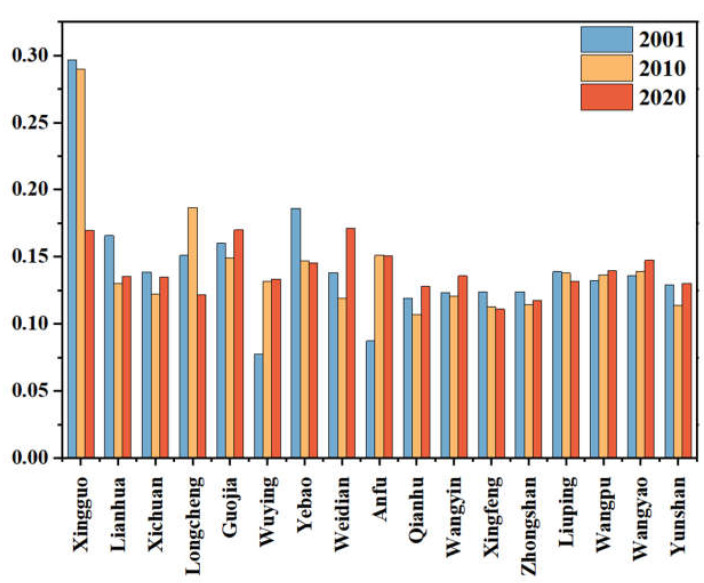
T The aging level of Qin’an County.

The population aging index showed slight fluctuations. The aging index of most villages and towns fluctuated less, and the overall aging phenomenon was relatively stable, as shown in Figure 4. Among them, Xingguo Town has the greatest fluctuation degree. Xingguo Town is the residence of the Qin’an County government. It has a relatively developed economy and a majority of the young and middle-aged population, so the aging phenomenon shows a decreasing trend year by year.

**Figure 5:**
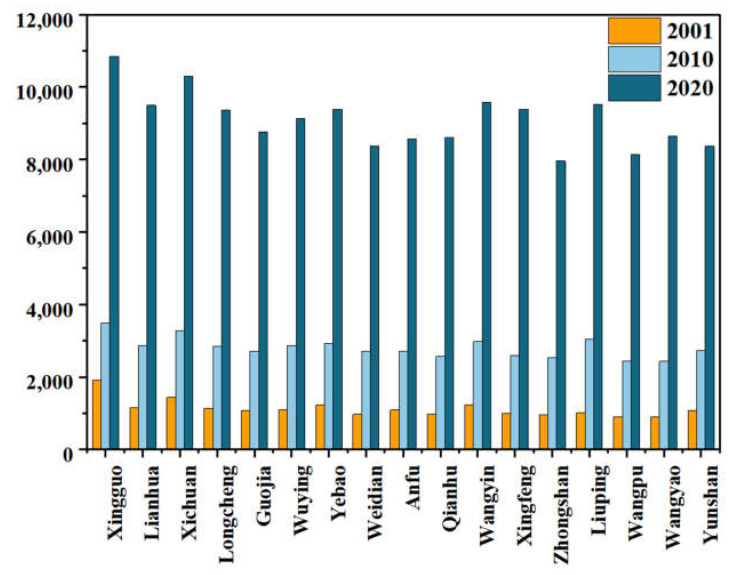
Per capita disposable income of Qin‘an County.

Qin’an County’s overall farmers’ disposable income was significantly increased. This is consistent with the rapid economic development of China. The average value increased from 1130 yuan in 2001 to 9086 yuan in 2020. The per capita disposable income in the south as a whole is higher than in the north, because the non-agricultural level of the labor force was higher in the south, and the overall income of the south was diversified, as shown in Figure 5.

### 5.2. Temporal and Spatial Evolution Characteristics of Agricultural Eco-Efficiency under Population Outflow

#### 5.2.1. Time Evolution Characteristics of Agricultural Eco-Efficiency

**Figure 6:**
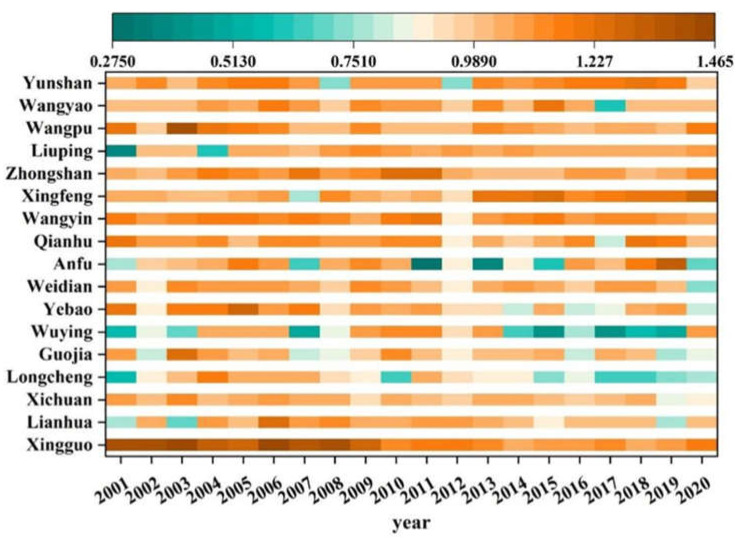
The evolution of agricultural eco-efficiency in Qin’an County.

Based on the SBM-Undesirable model, we measured the agricultural eco-efficiency of 17 villages and towns in Qin’an County from 2001–2020, as shown in Figure 6. The agricultural eco-efficiency of Qin’an County was mostly 1 or above. The agricultural eco-efficiency of Qin’an County had achieved effective development, and the input-output ratio of agricultural economic development and resource environment had reached a better state. By comparing the agricultural eco-efficiency values of 17 townships in Qin’an County, we found that the differences between regions first decreased, then increased, and then decreased over time. The overall change presented a slight fluctuation state, and the agricultural eco-efficiency tended to be stable with the evolution of time; this trend is in line with China’s 13th Five-Year Plan in 2016, which aims to ensure food security while being environmentally friendly. There were great differences between them at the township level. In 2001, the highest value of the difference between the towns was 1.4133, the lowest value was 0.3931, and the difference between the highest value and lowest value was 1.0202. In 2005, the difference between them was the smallest, and the effectiveness was the highest. The agricultural eco-efficiency of most towns in Qin’an County was greater than 1. This is mainly because China increased the policy of two exemptions and three subsidies in 2005, which greatly encouraged the enthusiasm of grain farmers and injected great vitality into agricultural development. The minimum efficiency value was in 2011, only 0.2784. This has much to do with China’s intensified monitoring of the rural environment in the same year. The highest value was in 2006, reaching 1.4613. However, with the evolution of time, the efficiency value of a small number of towns was less than 1. This indicates that it has not achieved effective development. Therefore, it is necessary to strengthen the technology and experience of the agricultural development of Qin’an County, thereby improving the agricultural eco-efficiency.

#### 5.2.2. The Spatial Distribution of Agricultural Eco-Efficiency

**Figure 7:**
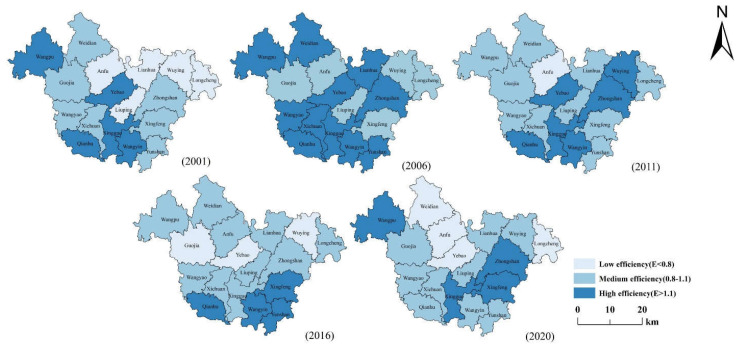
The spatial distribution of agricultural eco-efficiency in Qin’an County.

We calculated the agricultural eco-efficiency of 17 villages and towns in Qin’an County. The spatial distribution of agricultural eco-efficiency of villages and towns in 2001, 2006, 2016, and 2020 was plotted through ARCGIS 10.4 software, as shown in Figure 7.

In 2001, the agricultural eco-efficiency of most villages and towns in Qin’an County was at a lower level. The highest value was 1.4133, and the lowest value was 0.3931. Only a small number of townships were at a high level. The overall trend was high in the south and low in the north. This was because the overall terrain of Qin’an County was high in the north and low in the south. In 2006, the overall agricultural eco-efficiency of Qin’an County was slightly more than that in 2001. The highest value was 1.4613, and the lowest value was 1.0337. Qin’an County was at a higher level as a whole. The high level was mainly in the plain area, and the overall trend was still low in the north and high in the south. The overall level of Qin’an County in 2011 was slightly less than that in 2006. The overall value was at a higher level. The highest value was 1.2501, and the lowest was 0.2784. The high -value was in areas with low terrain. In 2016, although most of Qin’an County’s efficiency was at a higher level, there was only a slight increase in the value. The highest value was only 1.1547. The spatial distribution showed a trend of high south and low north. The agricultural eco-efficiency had been decreased from 2011 to 2016. The government had always paid attention to energy conservation and emission reduction during the “Twelfth Five-Year Plan” planning period, and reduced input in pesticide fertilizers, resulting in a reduction in efficiency. The agricultural eco-efficiency of Qin’an County in 2020 was slightly less than that in 2016, with the highest value of 1.2717. The overall level was at a lower level, and it had a spatial distribution of high in the south and low in the north. The overall agricultural eco-efficiency of Qin ’an County showed fluctuation increase. This is consistent with the government’s No. 1 document that focused on “agriculture, rural areas, and farmers” and “encourage the development of circular agriculture and ecological agriculture.” This shows that the government attaches great importance to the ability of sustainable agricultural development and avoids the decline of agricultural eco-efficiency.

### 5.3. Spillover Effect of Influencing Factors under Population Outflow

#### 5.3.1. Spatial Autocorrelation Test

This paper studied the spatial correlation of the agricultural eco-efficiency of 17 villages and towns in Qin’an County from 2001 to 2020 through the Stata15 software. The global Moran’s I index is shown in Table 3.

In Table 3, the global autocorrelation Moran’s index of agricultural eco-efficiency has passed the significance test of 10%, except for 2005, 2007, 2009, and 2019. This indicates that the agricultural eco-efficiency of Qin’an County has the characteristics of spatial autocorrelation. Moran’s index was positive in 2001–2003, 2008, 2018–2018, and 2020. This indicates that there was agglomeration in the spatial distribution of agricultural eco-efficiency. Moran’s index was negative in 2004–2007 and 2009–2010. This indicates that there was a diffusion in the spatial distribution. In addition, the Moran’s index values in 2001–2003, 2008, and 2011–2020 showed a fluctuation pattern of rising first, then falling, then rising, and then falling again. The agricultural eco-efficiency of Qin’an County showed a fluctuating agglomeration during this period. On the whole, the agricultural eco-efficiency of Qin’an County shows a spatial distribution of fluctuating agglomeration.

#### 5.3.2. The Spatial Panel Econometric Model Test

The spatial correlation test of agricultural eco-efficiency showed that they had significant spatial correlation. Therefore, geospatial elements need to be considered when studying the effects of agricultural eco-efficiency. This problem can be solved by the spatial panel econometric model. Based on existing research [29], it is necessary to first combine the Lagrange multiplier (LM) and robust Lagrange multiplier (robust LM) before the model estimation. It is more appropriate to judge the existence form of spatial correlation (in terms of error term or lag term), that is, SEM or SLM. Second, it is necessary to determine whether SDM can be simplified to SEM and SLM. The test shows that Moran’s I test reached a 0.01 significance level, and LRations, Walds, and LM-error tests reached 0.01 significance levels. The LM-lag test was significant at the 0.01 level. All are significant at the level of 0.01. The results of the Hausman test tend to use a fixed effect model, and the SDM model should be selected. The test results are shown in Table 4.

#### 5.3.3. Effect Decomposition

Since the SDM model contains both the explained variables and the spatial lag terms of the explanatory variables, the spatial lag of the explanatory variable has an impact on the feedback effect. Therefore, the estimated coefficients of the SDM model were only effective in the direction and significant. It cannot accurately reflect the extent of the explanatory variable’s impact on the interpretation variable. Therefore, we further estimated the direct effect, indirect effect, and total effect of the model based on the partial differential method proposed by Lesage [30]. The estimation results are shown in Table 5.

Influencing factors of local agricultural eco-efficiency: The direct effect of non-agricultural labor level on agricultural eco-efficiency was positive and significant at 10%. This indicates that the non-agriculturalization level of labor can slightly promote the improvement of local agricultural eco-efficiency. The non-agricultural transfer of labor can promote the input of agricultural technology, increase agricultural output and income, and promote the expected output. The direct effect of aging on agricultural eco-efficiency was positive and significant at 1%. This indicates that aging can significantly improve agricultural eco-efficiency. With the increase of the aging degree, the labor resources will be reduced, and the process of mechanization and intensive production will be accelerated. This is conducive to the improvement of agricultural eco-efficiency. The direct effect of per capita disposable income on agricultural eco-efficiency was positive and significant at 1%. This indicates that per capita disposable income can significantly improve the local agricultural eco-efficiency. The increase in disposable income led farmers to introduce mechanized tools to replace labor and accelerate agricultural production. The impact of land scale and irrigation index on agricultural eco-efficiency was positive, but the impact was not significant. This indicates that they have weak effects on agricultural eco-efficiency. The impact of the re-seeding index on the agricultural eco-efficiency was negative. However, it did not pass the signficance test, indicating that the impact on the agricultural eco-efficiency was weak. The impact of planting structure on agricultural eco-efficiency was positive and significant at 1%. This indicates that the increase in the area of crop planting is conducive to the improvement of agricultural eco-efficiency. The impact of the disaster rate on agricultural eco-efficiency was negative and significant at 5%. This indicates that the larger the disaster area, the less the agricultural output. This has an inhibitory effect on agricultural eco-efficiency. The impact of agricultural structure on agricultural eco-efficiency was positive and significant at 1%. This indicates that the agricultural structure can significantly improve agricultural eco-efficiency. The increase in agricultural income has driven the enthusiasm of labor for agricultural production and increased agricultural production, thereby improving agricultural eco-efficiency.

Space spillover effect of influencing factors: The estimated coefficients of indirect effects and total effects of the non-agricultural level of labor on agricultural eco-efficiency were positive but failed to pass the significance test. This indicates that the non-agriculturalization level of labor has a positive spillover effect on agricultural eco-efficiency, but the spatial spillover effect is not significant. This shows that the promotion ofnon-agricultural labor efficiency on agricultural ecological efficiency is mainly reflected through direct effects rather than spatial spillover effects, The estimated coefficient of the indirect effect of aging on agricultural eco-efficiency was positive, but it failed the significance test. This indicates that aging has positive spillover effects on agricultural eco-efficiency, but the spatial spillover effect is not significant. The estimated coefficient of total effect was 1.4124 and significant at 10%; that is, every 1% increase in the degree of aging promotes the growth of agricultural eco-efficiency by 1.4124%. This indicates that the promotion effect of aging on agricultural eco-efficiency was reflected through direct effects rather than spatial spillover effects. The indirect effect of farmers’ per capita disposable income on agricultural eco-efficiency was 0.0002 and significant at 1%. This indicates that the per capita disposable income has a significant positive spatial spillover on agricultural eco-efficiency. The indirect effects and total effects of land scale on agricultural eco-efficiency were positive and significant at 5%. This indicates that the impact of land scale on agricultural eco-efficiency was reflected through spatial spillover effects. The indirect effect of the irrigation index on agricultural eco-efficiency was negative, and the total effect was positive. Neither of them passed the significance test. This indicates that the impact of the irrigation index on agricultural eco-efficiency was through a direct effect. The indirect effect and total effect of the re-seeding index on agricultural eco-efficiency were negative and did not pass the significance test. This indicates that the spatial spillover effect of the re-seeding index on agricultural eco-efficiency was not significant. The indirect effect and total effect of planting structure on agricultural eco-efficiency were positive, but the indirect effect did not pass the significance test. The total effect was significant at 5%. The estimated coefficient of the indirect effect and the total effect of the disaster rate on the agricultural eco-efficiency were negative, but both did not pass the significance test. This indicates that the disaster rate has a negative spillover on agricultural eco-efficiency, but the spatial spillover effect is not significant. This indicates that the impact of the disaster rate on the agricultural eco-efficiency was reflected through the direct effect rather than the spatial spillover effect. The indirect effect and total effect of agricultural structure on agricultural eco-efficiency were positive, but only the total effect passed the significance test at the level of 10%. This indicates that the impact of agricultural structure on agricultural eco-efficiency was mainly through the direct effect.

### 5.4. Discussion

#### 5.4.1. The Influence Mechanism of the Driving Factor

(1) Direct effect

The factors in the agricultural development of Qin’an County under the population outflow mainly include labor, income, affected area, planting conditions, and agricultural structure.

With the transfer of labor, farmers’ income was significantly increased, and technological innovation and institutional innovation offset the negative impact of labor outflow, thereby adjusting the input of agricultural elements and increasing the overall agricultural output. In addition, the cash crops in Qin’an County have the characteristics of large areas and high efficiency. With the increase of the non-agricultural transfer ratio of labor, the agricultural output and efficiency of Qin’an County were increased, and the agricultural eco-efficiency was improved. The level of aging can significantly improve agricultural eco-efficiency [17]. The acceleration of aging can promote technological innovation and economic growth [16], thereby accelerating the process of agricultural modernization. Therefore, it is conducive to the improvement of agricultural eco-efficiency with the characteristics of “two-wheel drive.” The per capita disposable income affects the input of funds, technology, and labor. The per capita disposable income of Qin’an County has significantly increased over the past 20 years. The increase in income led farmers to increase their input in agricultural technology, thereby avoiding the reduction benefits from the lower labor costs. In addition, it can also motivate farmers to produce and sell agricultural products with higher prices, that is, to increase the production of cash crops, thereby improving agricultural eco-efficiency [8].

The improvement of land scale is conducive to the intensive and mechanized reform of agricultural production modes. This can improve agricultural eco-efficiency to a certain extent [16]. The larger the irrigation index, the more water can be provided for crop growth. Therefore, it is more conducive to the growth of crops, the output of crops can increase, and the appropriate irrigation method can reduce the investment in pesticides. Therefore, agricultural eco-efficiency can be improved [31]. Contrary to the degree of irrigation, the greater the degree of re-seeding, the lower the fertility of the soil. It is not conducive to agricultural production, resulting in an inhibitory effect. Qin’an County mainly planted cash crops, the probability of re-seeding was very low, and the degree of excavation of cultivated land was not increased, so the effect of Qin’an County was not significant. The increase in the planting area of cash crops is conducive to the improvement of agricultural eco-efficiency. Because cash crops have the advantages of short production cycles and high economic benefits, the increase of expected output can improve agricultural eco-efficiency to a certain extent. The larger the affected area, the lower the crop yield. In addition, it is necessary to increase the input of pesticides to reduce the impact of the reduction of crop income. However, it is also accompanied by negative effects, which will have a negative effect on agricultural eco-efficiency [8]. The planting industries account for a large proportion of agricultural production of villages and towns in Qin’an County, with a dominant position and relatively high GDP. This further stimulated farmers’ enthusiasm for production, thereby increasing agricultural eco-efficiency [32].

(2) The spatial spillover effect

**Figure 8:**
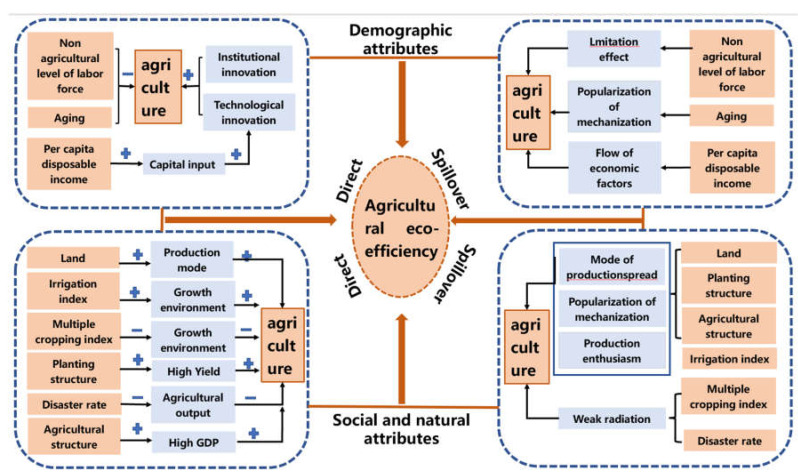
The driving mechanism of agricultural eco-efficiency from the perspective of population outflowing.

The promotion of a non-agricultural level of labor has led to an increase in income and a diversification of income. This has led to non-agricultural labor in neighboring or even the whole region and increased the spatial mobility of the labor force. This indicates that non-agricultural transfer of labor has radiation effects, affecting the decision-making of labor in the neighboring area. This can improve agricultural eco-efficiency. Aging has a positive spillover effect on agricultural eco-efficiency [17], but its indirect effect is not significant. Its total effect is a significant 10%. This indicates that the degree of aging can improve agricultural technology in the whole region, and then increase the popularization and investment level of agricultural mechanization for high benefits, thereby improving agricultural eco-efficiency. The per capita disposable income of farmers has a significant positive effect on the impact of agricultural eco-efficiency. This is consistent with the results in existing studies [33]. The flow of economic factors between regions can promote the economic development level of neighboring regions, thereby improving agricultural production conditions and agricultural eco-efficiency. The increase in the scale of land can promote agricultural production methods and the application of agricultural mechanization. The promotion and spread of production methods and mechanization have contributed to the improvement of agricultural eco-efficiency across regions. Therefore, the impact of cultivated land scale on agricultural eco-efficiency is mainly reflected through the spatial spillover effect. The spatial spillover effect of the re-seeding index on agricultural eco-efficiency was the negative effect and did not pass the significance test. This indicates that the spatial spillover effect of the re-seeding index on agricultural eco-efficiency was not significant. Cash crops took a large proportion of the agricultural production of most towns and villages in Qin’an County. There was no radiation effect between the planting methods, so the impact on neighboring townships and towns was not significant. However, the total effect was significant, indicating that the impact of planting structure on agricultural eco-efficiency was mainly reflected through direct effects. The estimated coefficient of indirect effect and the total effect of the disaster rate on the agricultural eco-efficiency were negative, but both of them did not pass the significance test. This indicates that the disaster rate had a not significant negative spillover effect on the agricultural eco-efficiency. This indicates that the impact of the disaster rate on the agricultural eco-efficiency was mainly reflected through the direct effect rather than the spatial spillover effect. The improvement of the agricultural structure indicates that the higher the proportion of agricultural income, the higher the expected output. This had a radiation effect on adjacent areas and stimulated the enthusiasm of adjacent areas to engage in agricultural production, thereby promoting the improvement of overall agricultural eco-efficiency (Figure 8).

#### 5.4.2. Policy

Based on the results of empirical analysis, this paper discussed policies for improving agricultural eco-efficiency from the following aspects.

(1) Encourage rural labor to return home and optimize the labor structure.

Due to the continuous increase in the labor cost of agricultural production from the reform and opening up, the rural labor force has been gradually transferred from the agricultural sector to the non-agricultural sector. This has changed the structure of the rural labor force and reduced the input of the agricultural labor force. However, this increased the input of mechanization, pesticides, and other chemical substances, such as pesticides. Although it promoted the improvement of agricultural eco-efficiency to a certain extent, it increased the emissions of carbon. Therefore, it is necessary to optimize and adjust the input structure of human factors, reasonably distribute family labor, and establish a mechanism to attract labor return, find a balance between rural labor and agricultural production. In addition, the government should promote technological progress and institutional innovation in agricultural production, improve crop yield and income while taking into account the ecological environment, and further improve agricultural eco-efficiency.

(2) Optimize the allocation of resource elements and strengthen agricultural ecological awareness.

The government should strengthen the impact of positive factors and promote the application of water-saving irrigation technology and scientific fertilization technology in agricultural production. In addition, we should pay attention to suppressing the impact of negative factors, realize precise input in chemical fertilizer, pesticides, etc., and reduce fertilizer consumption from the supply side [34] for resource saving. The government should carry out various forms of ecological environment protection theme activities to cultivate ecological awareness and low-carbon awareness of the rural population. Agricultural scientific research, extension institutions, and vocational training schools should help to train rural populations in production technology [35], improve their green production ideas, and promote the application of green technology in agricultural production.

(3) Improve the allocation of financial support to agriculture and increase input in agricultural research and development.

At present, funds related to agriculture are mainly from government finance and the income of farmers. Therefore, the government should promote the informatization of agricultural financial support for agriculture and guide the transformation of the structure of financial support for agriculture to the direction of agricultural ecological development. The government should reduce the subsidies for agricultural materials such as fertilizers and pesticides, increase the subsidies for environmental protection of agricultural materials, strengthen the development and promotion of ecological technology and the agricultural ecological compensation, and improve the utilization rate of agricultural financial funds in the agricultural field. In addition, it is necessary to strengthen cooperation and exchange with agricultural scientific research institutions and universities, increase input in research and development of agricultural production technology, develop more effective agricultural R&D and extension systems [6], develop more fertilizer substitutes, and pay attention to the development and extension of agricultural technology in combination with the concept of ecological civilization, so as to achieve rapid progress of agricultural technology and ecological development.

#### 5.4.3. Limitations

This paper conducted a relatively comprehensive study on the agricultural eco-efficiency of Qin’an County from the perspective of the population. There are some innovations, but there are still some limitations that need to be improved as follows: (1) This paper is based on the background of population outflow. In recent years, with the development of urbanization, the rural population has constantly been changing. It has impacts on all aspects of the countryside. The ecological environment is the national focus of China. Therefore, it is necessary to study the relationship between the two. In addition, this paper studied the villages and towns scale, which is more universal and referential than the large-scale study. This is beneficial to the follow-up agricultural development and the implementation of related agricultural policies. (2) This paper found that the relationship between the non-agricultural transfer of labor and agricultural eco-efficiency is linear. This is inconsistent with the U-shaped [7] of the existing research. Therefore, the relationship between labor and agricultural eco-efficiency varies from region to region, and this conclusion also enriches the existing research. However, the non-agricultural transfer of the labor force in Qin’an County is relatively stable. If the non-agricultural level of the labor force reaches a certain level, its relationship with ecological efficiency needs to be further verified. In future research, the regions with relatively high differences in the non-agricultural level of labor can be studied separately to draw more complete conclusions. (3) The research in this paper is mainly limited to three characteristics of the non-agricultural level of labor, aging, and income. The educational level of the rural population can affect agricultural production. However, due to the limited availability of data, there is no research about it. Therefore, it needs a more comprehensive analysis in the future.

## 6. Conclusions

This paper studied the agricultural eco-efficiency response from the perspective of population outflow. The conclusions of this paper are as follows: (1) The agricultural eco-efficiency of some villages and towns in Qin’an County has achieved effective development. The input-output ratio of agricultural economic development and resource environment has reached a relatively high level. The difference between different places first decreased, then increased, and finally decreased with time. The overall change shows a W-shaped fluctuation. The spatial distribution of overall agricultural eco-efficiency of Qin’an County shows a trend of low in the north and high in the south. This trend is still maintained with time. (2) The non-agricultural level of labor has a weak effect on the local agricultural eco-efficiency. The spillover effect is positive but did not pass the significance test. Aging can significantly improve the local agricultural eco-efficiency. The spillover effect of aging was positive, but the indirect effect was not significant. The total effect was significant at 10%. The disposable income of farmers can significantly improve agricultural eco-efficiency in the local and near and even the entire region. The direct effect of the land scale was positive, but the impact was not significant. The spillover effect was positive and significant at 5%. The direct effect of the irrigation index was positive, the indirect effect was negative, and the total effect was positive. It did not pass the significance test. The direct effects and spatial spillover effects of the re-seeding index were negative and did not pass the signficance test. The planting structure can slightly improve the local agricultural eco-efficiency and is significant at 1%. The spillover effect was positive. However, the indirect effect did not pass the significance test, and the total effect was significant at 5%. The agricultural structure can significantly promote local agricultural eco-efficiency. The spillover effect was the promotion effect, but only the total effect passed a significance test at 10%. The direct effect of the disaster rate was negative and significant at the level of 5%. The estimated coefficient of the spillover effect was negative, but none of them passed the significance test.

## Figures and Tables

**Table 1:** The input-output index of agricultural eco-efficiency.

Indicators	Variables	Variable Description	Units	Mean	Std. Dev.
Inputs	Land input	Total sown area of crops	10^3^ ha	4.2765	0.3047
	Agricultural labor	Agricultural practitioner	10^4^ person	1.1502	0.1125
	Mechanical input	Total power of agricultural machinery	10^4^ kw	1.2126	0.3742
		Fertilizer application amount	10^4^ tons	0.3649	0.0658
	Chemical input	Agricultural film application	10^4^ tons	0.0079	0.0027
		Pesticide usage	10^4^ tons	0.0152	0.0116
	Irrigation input	Effective irrigation area	10^3^ ha	5.7351	1.2070
Desirable output	Crop yield	Grain crop yield	tons	10,344.2697	2409.5690
		Economic crop yield	tons	27,378.8799	13,672.3308
	Agricultural output value	Total agricultural output value	10^8^ yuan RMB	0.8135	0.3755
Undesirable output	Carbon emission	Total carbon emissions from chemical fertilizers, pesticides, agricultural film, agricultural diesel, agricultural irrigation and agricultural sowing	10^4^ tons	0.4454	0.1212

**Table 2:** Variable settings.

Variable Types	Variable Name	Variable Description	Mean	Std. Dev.
Explained variable	Agricultural eco-efficiency	Super efficiency SBM model calculation	1.0272	0.0954
Explanatory variable	Non-agricultural level of labor force	Non-agricultural employment labor/labor force	0.3038	0.0533
	Per capita disposable income	Per capita disposable income	3905	2594
	Aging	The proportion of population 60 and above	0.1436	0.0115
Control variable	Land	Cultivated land area/total population	1.9093	0.0854
	Irrigation index	Effective irrigation area/total area of crop sowing	0.1147	0.0194
	Multiple cropping index	Federation area/crop sowing area	1.1045	0.1737
	Planting structure	Economic crop area/total area of crop sowing	0.2506	0.0178
	Agricultural disaster rate	The area of crop disaster/total sowing area	0.2846	0.0777
	Agricultural structure	Agricultural output value/total output value of agriculture, forestry, animal husbandry, and fishing	0.8271	0.0659

**Table 3:** Moran’s index of agricultural eco-efficiency.

Year	I	*p*-Value *	Year	I	*p*-Value *
2001	0.142	0.075	2011	0.061	0.070
2002	0.183	0.026	2012	0.150	0.061
2003	0.214	0.022	2013	0.094	0.050
2004	−0.261	0.034	2014	0.156	0.054
2005	−0.220	0.135	2015	0.276	0.006
2006	−0.258	0.035	2016	0.143	0.077
2007	−0.068	0.484	2017	0.207	0.025
2008	0.118	0.088	2018	0.284	0.004
2009	−0.230	0.110	2019	0.109	0.108
2010	−0.209	0.079	2020	0.258	0.013

Note: * indicates that at a level of 10%, the numbers in parentheses are the standard errors of each coefficient.

**Table 4:** Test results of spatial panel econometric model.

Testing Method	Statistics	*p*-Value	Testing Method	Statistics	*p*-Value
LM-spatial lag	28.620	0.000	Wald-spatial lag	27.74	0.0011
Robust LM-spatial lag	0.396	0.529	LR-spatial lag	33.65	0.0001
LM-spatial error	30.401	0.000	Wald-spatial error	38.54	0.0000
Robust LM-spatial error	2.178	0.140	LR-spatial error	43.02	0.0000

**Table 5:** Spatial effects of factors affecting agricultural eco-efficiency.

	Direct Effect	Indirect Effect	Total Effect
Non-agricultural level of labor force	0.1208 *	0.0494	0.1703
	(0.0689)	(0.1705)	(0.2069)
Aging	0.8044 ***	0.6080	1.4124 *
	(0.2732)	(0.6399)	(0.7925)
Per capita disposable income	0.0001 ***	0.0002 ***	0.0003 ***
	(0.0002)	(0.0001)	(0.0002)
Land	0.0174	0.0953 **	0.1126 **
	(0.0219)	(0.0480)	(0.0548)
Irrigation index	0.0543	−0.0311	0.0233
	(0.0570)	(0.1280)	(0.1578)
Multiple cropping index	−0.0461	−0.4638	−0.5100
	(0.2782)	(0.7350)	(0.8523)
Planting structure	0.5146 ***	0.4217	0.9363 **
	(0.1387)	(0.3678)	(0.4433)
Agricultural disaster rate	−0.1326 **	−0.0905	−0.0421
	(0.0665)	(0.1571)	(0.1845)
Agricultural structure	0.3705 ***	0.0865	0.4570 *
	(0.0669)	(0.2064)	(0.2367)

Note: *, **, *** indicate that at a level of 10%, 5%, and 1%, respectively, the numbers in parentheses are the standard errors of each coefficient.

## Data Availability

Data available on request due to restrictions e.g., privacy or ethical. The data presented in this study are available on request from the corresponding author. The data are not publicly available due to privacy.

## References

[1] Huang X., Xu X., Wang Q., Zhang L., Gao X., Chen L. (2019). Assessment of agricultural carbon emissions and their spatiotemporal changes in China, 1997–2016. Int. J. Environ. Res. Publ. Health.

[2] Ministry of Agriculture, National Development and Reform Commission, Ministry of Science and Technology National Agricultural Sustainable Development Plan (2015–2030). http://www.moa.gov.cn/sjzz/jgs/cfc/yw/201505/t20150528_4620635.htm.

[3] Nie W., Yu F.W. (2017). Research progress analysis of agricultural ecological efficiency. Chin. J. Eco-Agric..

[4] Rao J., Xu X., Ji X. (2011). Research on current situation, occurrence mechanism and Countermeasures of agricultural non-point source pollution in China. Agric. Econ. Issues.

[5] Guo Y., Zhou Y., Liu Y. (2020). Spatial and temporal evolution of rural population outflow in China and its driving mechanism. Geogr. Sci..

[6] Yang H., Wang X., Peng B. (2021). Agriculture carbon-emission reduction and changing factors behind agricultural eco-efficiency growth in China. J. Clean. Prod..

[7] Hou M., Yao S. (2018). Spatial spillover effect and threshold characteristics of the impact of China’s rural labor transfer level on agricultural ecological efficiency. Resour. Sci..

[8] Zhang Z., Liao X., Li C., Yang C., Yang S., Li Y. (2022). Spatial and temporal characteristics of county agricultural ecological efficiency in Hunan Province and its influencing factors. Econ. Geogr..

[9] Stępień S., Czyżewski B., Sapa A., Borychowski M., Poczta W., Poczta-Wajda A. (2021). Eco-efficiency of small-scale farming in Poland and its institutional drivers. J. Clean. Prod..

[10] Todorovic M., Mehmeti A., Scardigno A. (2016). Eco-efficiency of agricultural water systems: Methodological approach and assessment at meso-level scale. J. Environ. Manag..

[11] Heidenreich A., Grovermann C., Kadzere I., Egyir I.S., Muriuki A., Bandanaa J., Clottey J., Ndungu J., Blockeel J., Muller A. (2022). Sustainable intensification pathways in Sub-Saharan Africa: Assessing eco-efficiency of smallholder perennial cash crop production. Agr. Syst..

[12] Grassauer F., Herndl M., Nemecek T., Fritz C., Guggenberger T., Steinwidder A., Zollitsch W. (2022). Assessing and improving eco-efficiency of multifunctional dairy farming: The need to address farms’ diversity. J. Clean. Prod..

[13] Angelis-Dimakis A., Arampatzis G., Assimacopoulos D. (2016). Systemic eco-efficiency assessment of meso-level water use systems. J. Clean. Prod..

[14] Masuda K. (2016). Measuring eco-efficiency of wheat production in Japan: A combined application of life cycle assessment and data envelopment analysis. J. Clean. Prod..

[15] Liao J., Yu C., Feng Z., Zhao H., Wu K., Ma X. (2021). Spatial differentiation characteristics and driving factors of agricultural eco-efficiency in Chinese provinces from the perspective of ecosystem services. J. Clean. Prod..

[16] Li L., Xu W.X. (2021). Changes in agricultural ecological efficiency under the effect of rural population aging. J. South China Agric. Univ. Soc. Sci. Ed..

[17] Liang Y.W., Wang B.H. (2021). Study on the spatial-temporal evolution and influencing factors of an agricultural ecological efficiency in the Bohai Rim region. Ecol. Econ..

[18] Cao J.W., Zeng K. (2019). Research on agricultural ecological efficiency and influencing factors in the Yangtze River economic belt from the perspective of low carbon. Ecol. Econ..

[19] Hou M.Y., Deng Y.J., Yao S.B. (2021). Nonagricultural transfer of rural labor, fertilizer application intensity and agricultural ecological efficiency: Interaction and spatial spillover. Agric. Technol. Econ..

[20] Pan D., Ying R.Y. (2013). Evaluation method and demonstration of agricultural eco-efficiency in China: Analysis of SBM model based on undesired output. Acta Ecol. Sin..

[21] Coluccia B., Valente D., Fusco G., De Leo F., Porrini D. (2020). Assessing agricultural eco-efficiency in Italian Regions. Ecol. Indic..

[22] Yang B., Zhang Z., Wu H. (2022). Detection and attribution of changes in agricultural eco-efficiency within rapid urbanized areas: A case study in the Urban agglomeration in the middle Reaches of Yangtze River, China. Ecol. Indic..

[23] Ma L., Zhang W., Wu S., Shi Z. (2022). Research on the Impact of Rural Population Structure Changes on the Net Carbon Sink of Agricultural Production-Take Huan County in the Loess Hilly Region of China as an Example. Front. Environ. Sci..

[24] Tone K. (2002). A slacks-based measure of super-efficiency in data envelopment analysis. Eur. J. Oper. Res..

[25] Anselin L. (1988). Spatial econometrics: Methods and models. Stud. Oper. Reg. Sci..

[26] LeSage J.P., Robert P.K. (2009). Introduction to Spatial Econometrics.

[27] Liu D., Zhu X., Wang Y. (2021). China’s agricultural green total factor productivity based on carbon emission: An analysis of evolution trend and influencing factors. J. Clean. Prod..

[28] Parman J. (2012). Good schools make good neighbors: Human capital spillovers in early 20th century agriculture. Explor. Econ. Hist..

[29] Elhorst J.P. (2014). Matlab Software for Spatial Panels. Int. Reg. Sci. Rev..

[30] Lesage J.P. (2008). An introduction to spatial econometrics. Rev. D’Économie Ind..

[31] Liu Y., Zou L., Wang Y. (2020). Spatial-temporal characteristics and influencing factors of agricultural eco-efficiency in China in recent 40 years. Land Use Policy.

[32] Zhao J., Dang G., Tang X. (2022). Study on the spatial-temporal difference and influencing factors of agricultural ecological efficiency in China based on SBM Tobit model. J. Southwest For. Univ. Soc. Sci..

[33] Wu G., Riaz N., Dong R. (2022). China’s agricultural ecological efficiency and spatial spillover effect. Env. Dev Sustain..

[34] Long X., Luo Y., Sun H., Tian G. (2018). Fertilizer using intensity and environmental efficiency for China’s agriculture sector from 1997 to 2014. Nat. Hazards.

[35] Xiang H., Wang Y.H., Huang Q.Q., Yang Q.Y. (2020). How Much Is the Eco-Efficiency of Agricultural Production in West China? Evidence from the Village Level Data. Int. J. Env. Res. Pub. Health.

